# Dystroglycan versatility in cell adhesion: a tale of multiple motifs

**DOI:** 10.1186/1478-811X-8-3

**Published:** 2010-02-17

**Authors:** Chris J Moore, Steve J Winder

**Affiliations:** 1 Department of Biomedical Science, University of Sheffield, Firth Court, Western Bank, Sheffield, S10 2TN, UK

## Abstract

Dystroglycan is a ubiquitously expressed heterodimeric adhesion receptor. The extracellular α-subunit makes connections with a number of laminin G domain ligands including laminins, agrin and perlecan in the extracellular matrix and the transmembrane β-subunit makes connections to the actin filament network via cytoskeletal linkers including dystrophin, utrophin, ezrin and plectin, depending on context. Originally discovered as part of the dystrophin glycoprotein complex of skeletal muscle, dystroglycan is an important adhesion molecule and signalling scaffold in a multitude of cell types and tissues and is involved in several diseases. Dystroglycan has emerged as a multifunctional adhesion platform with many interacting partners associating with its short unstructured cytoplasmic domain. Two particular hotspots are the cytoplasmic juxtamembrane region and at the very carboxy terminus of dystroglycan. Regions which between them have several overlapping functions: in the juxtamembrane region; a nuclear localisation signal, ezrin/radixin/moesin protein, rapsyn and ERK MAP Kinase binding function, and at the C terminus a regulatory tyrosine governing WW, SH2 and SH3 domain interactions. We will discuss the binding partners for these motifs and how their interactions and regulation can modulate the involvement of dystroglycan in a range of different adhesion structures and functions depending on context. Thus dystroglycan presents as a multifunctional scaffold involved in adhesion and adhesion-mediated signalling with its functions under exquisite spatio-temporal regulation.

## Introduction

**Figure 1:**
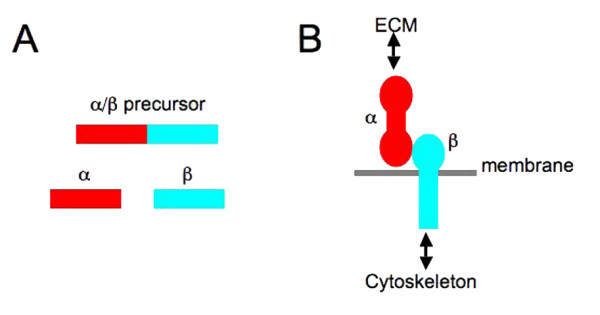
**A, a simple schematic representing the α-/β-dystroglycan pro-protein and subsequent cleavage to α- and β-dystroglycan**. B. Organisation of α- and β-dystroglycan at the cell membrane showing topology and major interactions.

Dystroglycan was first described as laminin binding protein from brain [1-3] and also identified as a glycan component of the dystrophin glycoprotein complex (DGC) of skeletal muscle, whence it derived its most commonly used name: dystroglycan [4]. Dystroglycan comprises two glycoproteins that are post-translationally cleaved from a single gene (Figure 1A). The extracellular peripheral membrane subunit α-dystroglycan undergoes extensive N- and O-linked modifications. The central mucin-like central region is particularly important for interactions between α-dystroglycan and laminin G (LG) module-containing extracellular matrix proteins such as agrin, perlecan and laminin itself (reviewed in [5]). The β-dystroglycan subunit is subject to some N-linked glycosylation and is a type 1 transmembrane glycoprotein binding to the carboxy-terminal domain of α-dystroglycan on the extracellular face, and to actin either directly or indirectly through one of a number of actin binding proteins, on its intracellular face (Figure 1B). Early hypotheses concerning the role of dystroglycan were influenced by its central role in the DGC of skeletal muscle and the interaction with the spectrin family protein dystrophin (reviewed in [6]). Mutations in the DMD gene leading to a complete absence of the dystrophin protein product give rise to the fatal X-linked condition Duchenne muscular dystrophy (DMD). The aetiology of DMD, with its sarcolemmal damage and necrosis; coupled with the domain structure of dystrophin that resembled spectrin led to the idea that dystrophin in concert with the DGC performed some sort of shock absorber role providing mechanical stability to the sarcolemma to withstand the forces of contraction and relaxation. This was envisaged to be in a similar way to that in which spectrin in the erythrocyte sub-membranous cytoskeleton provided visco-elastic support to the red blood cell membrane to withstand deformation during passage through small capillaries see [7] for a recent review. Whilst this is undoubtedly *part *of the role of dystrophin, dystroglycan and the DGC, it is almost certainly not the only role, and there is now a considerable body of evidence pointing to signalling functions for the DGC and dystroglycan in particular (see below). Other emerging concepts are that dystroglycan may not be an obligate heterodimer, in particular β-dystroglycan may have functions in the absence of α-dystroglycan: functions in cell polarity, in the nucleus or in cancer [8-10].

## Dystroglycan in disease

**Figure 2:**
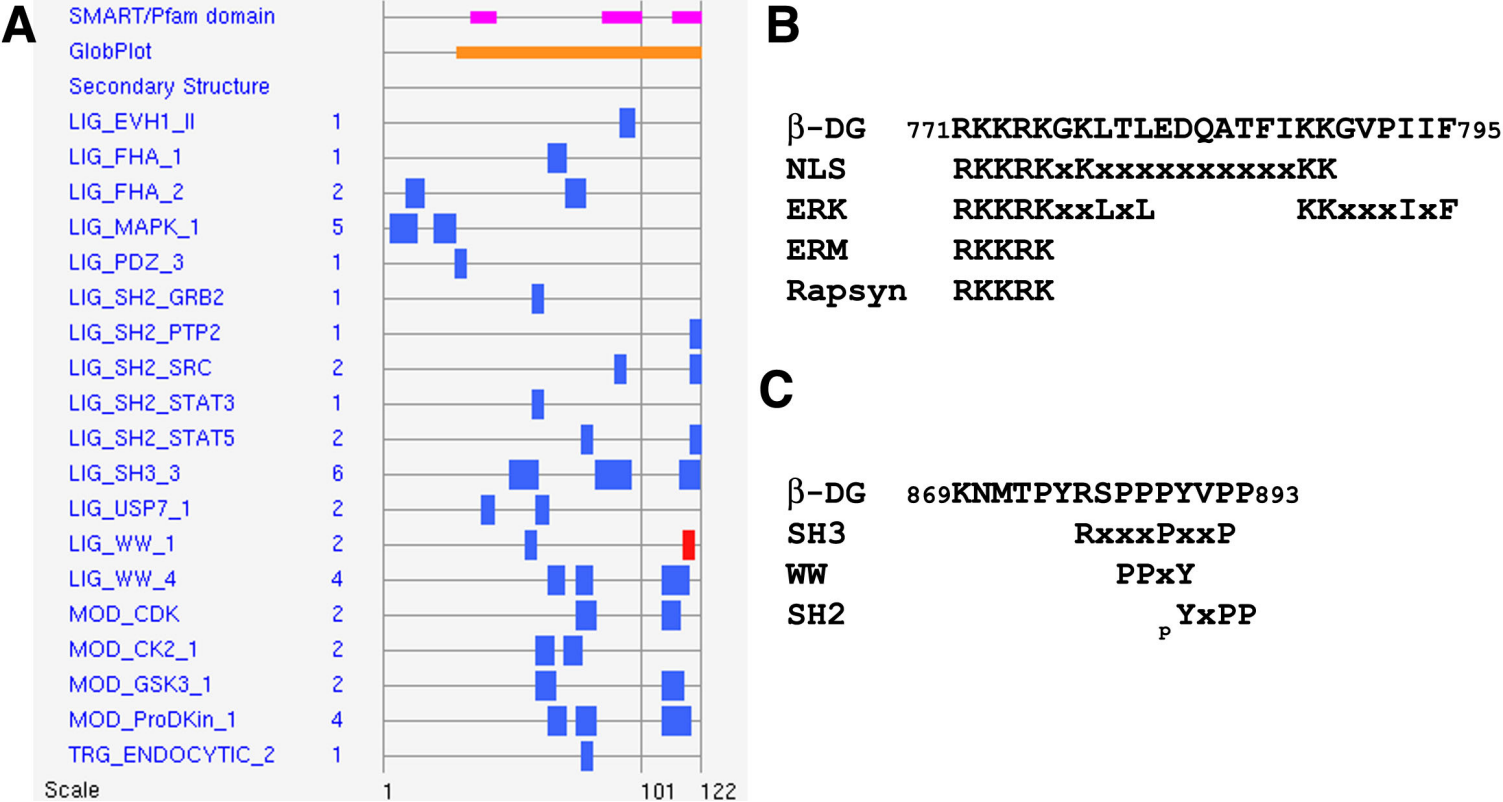
**A. Summary of ELM output for β-dystroglycan cytoplasmic domain**. Horizontal grey lines represent the primary sequence of the dystroglycan cytoplasmic domain, pink bars are low complexity and orange bars are disordered regions, blue and red boxes represent the position(s) of specific linear motifs as described on the left. Numbers represent the number of occurrences of each motif in the sequence, full annotation can be found at http://www.elm.eu.org. B Scheme showing detail of sequence and position of interaction sites in the β-dystroglycan juxtamembrane region including Rapsyn, ERM, ERK and NLS. C Scheme showing detail of sequence and position of interaction sites in the β-dystroglycan carboxy terminus including WW SH3 and SH2. X denotes any residue within the ELM consensus, and pY represents phosphorylated tyrosine.

We have sought over the last 10 years to unravel some of the signalling mechanisms associated with the DGC and with β-dystroglycan in particular. Within the 120 residue cytoplasmic region of β-dystroglycan, comprising almost 25% proline, are over 40 predicted interaction sites comprising 19 different functional motifs (ELM prediction [11], Figure 2A). Whilst many of these remain to have ligands or functions ascribed, two particular clusters of overlapping motifs, one spanning the extreme C-terminal 6 amino acid residues of β-dystroglycan, and the other covering the 10 juxtamembrane residues of the cytoplasmic region (Figure 2B, C) have received particular attention. In the following pages we will focus on the regulation and consequences of these overlapping motifs for dystroglycan function in the cell. However in order to place some of these findings in context it is worth considering the broader role of dystroglycan in disease and signalling related to such diseases.

As a cell adhesion molecule dystroglycan performs two basic functions: as a physical connection between extracellular matrix and cytoskeleton, and as a transducer of signals from outside to inside, so called 'outside in signalling' (see below). Whatever ones belief in the contribution of either of these functions to normal or disease processes, it remains difficult to dissect functionally the mechanical adhesion component from the signalling component, indeed they probably are inseparable. Nonetheless different aspects of disease tend to be ascribed to mechanical or signalling functions. As alluded to above, a mechanical role for the DGC in muscle, of which dystroglycan is an essential core element, initially held sway, but this has been supplanted to a certain extent by signalling roles (reviewed in [12-14]). We shall discuss some of the signalling function that is directly associated with dystroglycan in later sections, but it is also worth noting that the DGC as a whole acts as a signalling scaffold for pathways that are not directly mediated through dystroglycan. Examples of these include nitric oxide synthase (NOS) signalling, Pi3-kinase and Akt-mediated survival signalling and pathways involving small and heterotrimeric G-proteins, mediated variously through dystrophin, dystrobrevin and syntrophins, for examples see [15-19]. Although in muscle there appears to be only one DGC, there must be spatio-temporal regulation of these signalling functions through mechanisms that remain to be elucidated, as it seems unfeasible that they could all operate simultaneously through the same complex. But besides these signalling roles that would clearly be perturbed if the integrity of the DGC were compromised due to changes in dystroglycan, there are more direct changes in dystroglycan function that would intuitively appear to have a more mechanical function. Disease associated with this latter scenario are termed the dystroglycanopathies, and arise not through mutations in dystroglycan itself, but in a number of genes that are known or presumed to have function in the glycosylation of dystroglycan [[20][Muntoni, 2007 #2825]]. These glycosyltransferase genes are responsible for the essential post-translational modifications of α-dystroglycan that are required for LG module binding, and without this glycosylation, dystroglycan is functionally unable to connect to the extracellular matrix. This can result in very severe disease involving both muscle and neurological phenotypes such as in Fukuyama and congenital muscular dystrophy 1D, muscle eye brain disease or Walker Warburg syndrome. These phenotypes also highlight another area of significant involvement of dystroglycan function, namely in the neuronal system, with functions in neuronal cell migration and patterning as well as neuronal cell adhesion, brain architecture and neuronal signalling. A discussion of this is beyond the scope of this review and readers are directed to more authoritative sources [21].

## Dystroglycan as an adhesion receptor in muscle and non-muscle tissues: the carboxy-terminal region

**Figure 3:**
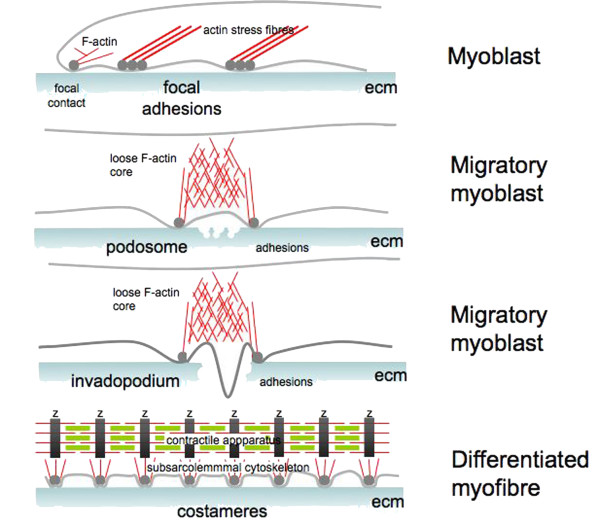
**Cartoon of adhesion types found in myoblasts and myofibres**. Grey represent the cell membrane, red represents actin filaments, green myosin filaments, dark grey balls dystroglycan containing adhesions, black bars labelled Z are the Z-discs of striated muscle and the extracellular matrix is represented by the blue-grey gradient at the bottom.

Dystroglycan is ubiquitously expressed and consequently found to have critical roles in a variety of situations where cell adhesion is important. In development, the laminin binding activities of dystroglycan are crucial for early embryonic basement membrane formation, to the extent that mice null for dystroglycan fail to properly assemble Reichert's membrane, the first extra-embryonic basement membrane, resulting in a failure in implantation and early embryonic lethality [22]. Branching epithelial morphogenesis in lung and kidney, and probably other glandular epithelial tissues that form by a similar process, is also reliant on dystroglycan function (reviewed in [23,24]) as is the development and maintenance of many neuronal structures. Various clinical conditions and experimental and model systems have revealed roles for dystroglycan in neuronal migration, the blood brain barrier, retinal layering, Schwann cell wrapping and the architecture of the nodes of Ranvier [21,25]. Notwithstanding the ubiquitous distribution and multiple roles in multiple tissues, it remains that dystroglycan has been studied most extensively in muscle, and in particular in relation to its role in the dystrophin glycoprotein complex and associated muscular dystrophies. From the point of view of muscle, dystroglycan has a role in all stages of muscle development, and is clearly identified in the developing myotome, as well as in migratory myoblasts [26] where it plays an important role in organising laminin 111 [27]. A role for dystroglycan in adhesion in migratory myoblasts and more mature 'static' myofibres highlights the versatility of this adhesion receptor and points to multiple modes of interaction and regulation. One commonly used mechanism for regulating adhesion-dependent processes is tyrosine phosphorylation. Tyrosine phosphorylation of β-dystroglycan in response to cell adhesion has been recognised for some time as an important molecular switch to govern dystroglycan function in relation to dystrophin and utrophin binding [8,28]. This concept has been further extended recently by the identification of phospho-tyrosine mediated regulation of β-dystroglycan function in both podosomes and focal adhesion structures (Figure 3), in addition to its established function in the costameric adhesion structures of skeletal muscle [13].

A number of studies utilising non-muscle cells, myoblasts and myotubes had identified tyrosine 890 of β-dystroglycan (Y890 in mouse, Y892 in humans) as a target for phosphorylation by Src and other Src family kinases, and furthermore that Y890 phosphorylation regulates the interaction between dystroglycan and key cellular binding partners, most notably dystrophin and utrophin [28-32]. Considering dystroglycan as a cell adhesion molecule, with regulation by tyrosine phosphorylation; we have investigated dystroglycan function in light of the integrin paradigm. Binding of extracellular matrix proteins to integrins causes allosteric changes across the plasma membrane resulting in the binding of signalling and adaptor proteins, leading to tyrosine phosphorylation and the establishment of multiprotein signalling complexes on the intracellular face, so called outside-in signalling (see [33] for recent review). Integrins constitute a large family of cell adhesion receptors found in many cell types and tissues. Integrins make connections with extracellular matrix proteins and cytoskeletal components, and they are all capable of signalling via tyrosine kinases (reviewed in [34,35]). Depending on the cell, the extracellular matrix ligand and the particular combination of α-/β-integrin heterodimers, different signalling complexes are assembled leading to different intracellular responses. Unlike the multitude of integrin heterodimer combinations with different cell and tissue distributions however, there is only one ubiquitously expressed α-/β-dystroglycan heterodimer. Therefore integrins are represented in multiple genes giving rise to multiple functions, whereas dystroglycan is represented by one gene but is still capable of mediating multiple functions. In the case of dystroglycan in particular we have focussed our attentions on the cytoplasmic domain of β-dystroglycan and the myriad of predicted protein-protein interaction sites that could be involved in mediating these multiple functions (Figure 2). We have therefore investigated dystroglycan function and the potential interactions mediated through these sites in the context of adhesion-mediated signalling. Dystroglycan engagement by laminin results in the phosphorylation of the cytoplasmic region of β-dystroglycan with the site being unequivocally identified as tyrosine 890 [29,31]. Not only was dystroglycan a substrate for adhesion-mediated tyrosine phosphorylation in a manner analogous to integrins, but also the tyrosine phosphorylation of dystroglycan at Y890 resulted in the disruption of the C-terminal WW domain interaction site for both utrophin and dystrophin (Figure 2C) [28-30]. Interestingly however, caveolin-3 binding to this same site via a WW-like domain [36] appeared insensitive to the tyrosine phosphorylation [28]. Therefore extracellular signals were sufficient to generate a phosphotyrosine signal which acts as a molecular switch subsequently altering the binding of dystroglycan to two of its major ligands connecting it to the actin cytoskeleton, no different to outside-in signalling. The kinases responsible for the phosphorylation of dystroglycan was subsequently demonstrated to be Src and other Src family members [31], also key integrators of integrin-mediated adhesion signalling. In addition to the interaction between dystroglycan and dystrophin or utrophin, other cytolinker proteins such as plectin have also been demonstrated to make interactions with dystroglycan, and may also have a compensatory and protective role in situations where dystroglycan function is partly compromised such as in the *mdx *mouse [37]. Whether there is direct competition for binding to dystroglycan between plectin and dystrophin *in vivo *is not clear, but biochemically, binding sites on dystroglycan for the two proteins do overlap, and in the absence of dystrophin, such as in the *mdx *mouse, the organisation of plectin and dystroglycan in the sarcolemma does change [37]. A further competitive interaction is that between the signalling adapter Grb2 and dystrophin. Grb2 binds to the SH3 domain interaction motif (PxxP) that overlaps the WW domain binding motif (PPPY; Figure 2C) in dystroglycan [38-40], and although at the biochemical level the interaction is clearly competitive, the functional significance of any Grb2 interaction with dystroglycan in cells and tissues remains unclear. From studies in Drosophila there appears also to be competitive interactions for dystroglycan, but rather than the C-terminal WW domain motif being the focus of interactions a non-overlapping SH3 motif located more amino-terminally is functionally more important for such functions as polarity determination [41]. Nonetheless the WW domain interaction site is essential, though due to the presence of a second WW domain interaction site that can also mediate interactions through dystroglycan, is partially redundant [42]. It should be noted however, that compared to the almost 95% identity between vertebrate dystroglycan cytoplasmic regions, Drosophila dystroglycan is considerably more divergent [43].

Antibodies directed against α-dystroglycan and β-dystroglycan readily recognise both proteins in the sarcolemma and more precisely in costameres of striated muscles. However in the analogous focal adhesion structures of fibroblast or myoblast cells for example, only the extracellular epitopes of α-dystroglycan are detectable by antibody staining [44,45]. As α- and β-dystroglycan are believed to be obligate heterodimers it has therefore been assumed that whilst β-dystroglycan was not detectable in focal adhesions by antibody staining or using GFP-fusions, that it is nonetheless present. It has been hypothesised that the cytoplasmic region of β-dystroglycan is in an environment subject to considerable 'molecular crowding' or 'epitope masking', a phenomenon seen with other focal adhesion proteins such as α-actinin [46], rendering it undetectable by routine visualisation techniques. Despite its 'presumed presence' the inability to detect or visualise dystroglycan in other classes of adhesion has hampered further investigation in this area. Recently significant inroads have been made into this problem, with the finding that dystroglycan is a component of podosome adhesion structures, and techniques have been developed to visualise dystroglycan in focal adhesions making the study of dystroglycan in these adhesion types more amenable [47,48].

From work investigating the role of dystroglycan in adhesion structures it is clear that DG can play a part in regulating the type and abundance of such structures within a cell. Over-expression of tagged dystroglycan results in a relative increase in the levels of focal complexes and a decrease in fibrillar adhesions compared to other cellular adhesions whereas shRNA knockdown of dystroglycan causes the reverse effect [48]. The mechanism of action of dystroglycan in this case is not known, but a candidate to mediate this effect may be vinexin recruitment. Dystroglycan has been shown to interact with vinexin, a vinculin binding partner that resides in focal adhesions. This binding occurs between the 3rd SH3 domain of vinexin and the proline rich C-terminal region of β-dystroglycan, specifically the most C-terminal SH3 binding domain [48] (Figure 2C). Dystroglycan shRNA knockdown myoblast cells spread poorly on E3 laminin (a dystroglycan specific ligand), but can be rescued through overexpressing dystroglycan. However, overexpression of a construct mutated in the vinexin-binding site is unable to rescue this cell spreading defect. Therefore, the interaction between vinexin and dystroglycan, and possibly vinculin too, can modulate cell spreading. The interaction of a vinexin SH3 domain with the C-terminal SH3 motif in dystroglycan also highlights a further potential competitive interaction. Peptide SPOT array data indicated that the vinexin SH3 interaction with dystroglycan was sensitive to Y890 phosphorylation [48], just as the binding of utrophin or dystrophin to this region are prevented by tyrosine phosphorylation [29,30]. However as both vinexin and utrophin bind the same region when not phosphorylated, there could be direct competition between the two proteins for the SH3 motif to which vinexin binds and the overlapping type 1 WW domain motif to which utrophin binds (dystrophin is not present in myoblasts; see Figure 2C), rather than Src modulated phosphorylation-dependent switching for this site.

**Figure 4:**
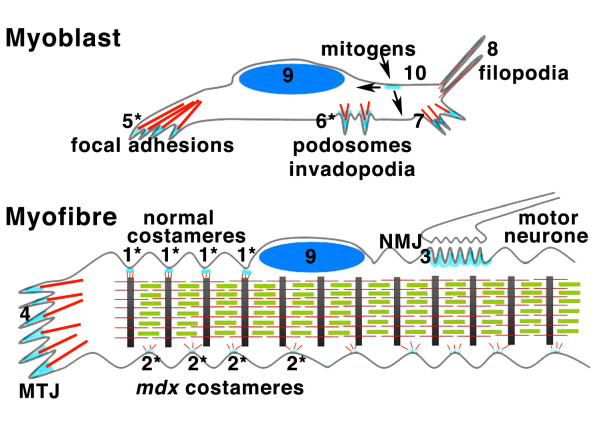
**Cartoon summary of different dystroglycan functions/complexes in myoblasts top or myofibres bottom referred to in the main text**. Grey represent the cell membrane, red represents actin filaments, green myosin filaments, black Z-discs of striated muscle, dark blue ellipse is the nucleus and all pale blue represents different dystroglycan-containing structures according to the numbers. 1, normal costameric adhesions containing the dystrophin-dystroglycan glycoprotein complex. 2, *mdx *costameric adhesions with compromised connectivity to the underlying z-discs, containing plectin-dystroglycan and utrophin-dystroglycan complexes. 3. Neuromuscular junction (NMJ) containing; dystrophin-dystroglycan glycoprotein, utrophin-dystroglycan and rapsyn dystroglycan complexes 4. Myotendinous junction (MTJ) containing utrophin-dystroglycan complex. 5. Focal adhesions containing dystroglycan vinexin and possibly dystroglycan utrophin complexes. 6. Podosomes and invadopodia containing dystroglycan Tks5 and dystroglycan-Tks5-Src complexes. 7. Focal contacts containing dystroglycan vinexin complexes. 8. Filopodia containing dystroglycan-ezrin and dystroglycan-ezrin-Dbl complexes. 9. Nuclear dystroglycan in complex with other dystrophin associated proteins including Dp71. 10. Dystroglycan ERK complexes directing ERK signalling to nucleus or cytoplasm. * represents the possibility for regulation of the complex at this site by tyrosine phosphorylation.

Another adhesion type where dystroglycan has recently been found to play a role is the podosome. In podosomes however, there does appear to be Src-mediated Y890 phosphorylation-dependent switch [47]. Podosomes are a less dense arrangement of adhesion proteins around an actin core (Figure 3), most often observed in normally migratory cells and invasive cells including tumour cells. The looser arrangement of proteins in the podosome may have facilitated the accessibility of dystroglycan antisera making the visualisation of dystroglycan in podosomes more straightforward than in focal adhesion like structures. Moreover the observation that dystroglycan in podosomes is phosphorylated on Y890 also precludes some of the binding partners that might occupy the C-terminal residues, such as vinexin or utrophin for example. However, Y890 phosphorylation does then generate an SH2 domain ligand [31], to which Src itself can bind [31,47] along with which it recruits Tks5 to podosomes [47]. In an analogous manner to dystroglycan in focal adhesions, the levels of dystroglycan in the cell and the switching of dystroglycan function, in this case by Src-mediated phosphorylation of Y890 to generate a ternary dystroglycan:Tks5:Src complex, can regulate the formation of podosomes in myoblasts [47] (Figure 4). Accordingly, dystroglycan in concert with integrins, is involved in the spatial and temporal remodelling of adhesion numbers, types and distribution. Through this dystroglycan therefore plays an important role in effecting changes in myoblast anchorage, motility and migration and consequently facilitates the differentiation process and the establishment of costameric adhesion structures in mature myofibres.

## The juxtamembrane region: Rapsyn, ERM proteins, ERK signalling and nuclear localisation

Transient overexpression of dystroglycan-GFP in the majority of cells in culture is associated with the appearance of numerous filopodia and microvilli like structures. Concomitant with the formation of these structures is a dramatic rearrangement of the actin cytoskeleton from the typical orthogonal array of stress fibres to a more peripheral band of actin cables and actin-containing microvilli and ultimately in the most extreme cases an apparent total dissolution of the stress fibre network and the appearance of numerous fine filopodia and dorsal microvilli-like structures rich in dystroglycan-GFP [49]. Biochemical analyses of the dystroglycan cytoplasmic domain in isolation, revealed that it had intrinsic actin binding and bundling properties, which could in part account for the ability of dystroglycan to induce the formation of actin bundles at the cell membrane and drive filopodia formation [49]. A series of deletion constructs of dystroglycan which removed all of the major domains of α- or β-dystroglycan in turn and in combination, revealed that the cytoplasmic domain of β-dystroglycan was both necessary and sufficient for filopodia formation providing that it was targeted to the plasma membrane either by secreted alkaline phosphatase-tags or by myristoylation sequences targeting it to the inner membrane leaflet [49,50].

A manual search of the sequence of the cytoplasmic domain of dystroglycan revealed a stretch of basic residues in the juxtamembrane region (Figure 2B) that resembled similar sequences in a number of other cell adhesion receptors (CD44, ICAM-1, ICAM-2 and L-selectin) that are able to interact with ezrin, radixin, moesin (ERM) family proteins [51-54]. In most cases the recruitment of activated ezrin to these cell adhesion receptors has been reported to induce the formation of microvilli structures [51,53,54], dystroglycan would also appear to be no exception in this regard. Deletion or alteration of the character of these sequences in dystroglycan was sufficient to prevent the association of ezrin with dystroglycan and prevented dystroglycan-induced actin-rich protrusions [50,55]. The co-localisation of endogenous dystroglycan and ezrin in microvilli-like structures and co-immunoprecipitation and GST-pulldown of dystroglycan and ezrin from cell extracts suggest a direct role for dystroglycan in the formation of microvilli-like structures [49,55]. Moreover, the dystroglycan-dependent and ezrin-dependent formation of filopodia also required the activation of Cdc42, as dominant negative Cdc42 prevented the dystroglycan-dependent microvilli formation and the dystroglycan-dependent recruitment of ezrin to the actin cytoskeleton [49,55]. Depletion of dystroglycan levels by RNAi also had an inhibitory effect on the ability of active Cdc42 to induce filopodia, indicating a central role for dystroglycan in the formation of filopodia in response to Cdc42 [50].

ERM family proteins have been demonstrated previously to interact with components of the Rho GTPase signalling machinery, notably RhoGDI [56] and the GDP/GTP exchange factor (GEF) for Cdc42 and Rho: Dbl [57,58]. An interaction between dystroglycan, ezrin and regulatory elements of the Rho GTPase signalling pathway could explain the actions of dystroglycan on filopodia formation. We identified a dystroglycan-Dbl-ezrin complex by a combination of immunoprecipitation, tagged protein pulldown and immunofluorescence microscopy. Furthermore we demonstrated that the targeting of a dystroglycan-Dbl-ezrin complex to the membrane promoted the formation of filopodia and microvilli by the local activation of Cdc42 at the membrane. Mislocalisation of the dystroglycan-Dbl-ezrin complex to the cytoplasm by using a cytoplasmic dystroglycan construct had a dominant-negative effect on filopodia formation [50]. Thus the peripheral membrane localisation of dystroglycan is again essential for its function as a scaffold for components of the actin-signalling machinery to productively generate new actin-based structures such as filopodia and microvilli which could also be important prerequisites for adhesion formation.

In addition to being a substrate for adhesion-mediated signalling, dystroglycan was also found to have an antagonistic role in other signalling cascades mediated by integrins. Most notably it has been demonstrated that integrin α6β1 and dystroglycan have antagonistic roles in signalling to the Ras-Raf-MEK-ERK (ERK) pathway, with dystroglycan having an inhibitory effect on integrin-mediated activation of the ERK pathway [59]. Dystroglycan function is also required, independently of integrin function, for laminin 311 mediated stretch-activated ERK signalling in alveolar epithelial cells [60]. The dystroglycan cytoplasmic region has predicted ERK MAPK binding sequences in the juxtamembrane region (Figure 2C) and through a combination of yeast-two hybrid and proteomic analyses components of the ERK pathway were found to interact with the cytoplasmic region of β-dystroglycan; both MEK and activated ERK were found as interactors. The activity of neither protein was affected by binding to dystroglycan, nor was dystroglycan a substrate, but surprisingly the localisation of dystroglycan with MEK or active ERK was quite different within the cell [45]. Active ERK localised to focal adhesions as had been demonstrated previously [61], where it colocalised with α-dystroglycan, whereas MEK was colocalised with β-dystroglycan in membrane ruffles [45]. This differential spatial localisation of two components of a canonical signalling cascade could provide a potential explanation for the antagonistic role of dystroglycan in opposing integrin-mediated ERK activation as above. A further development of this idea has been put forward, whereby scaffolds such as dystroglycan are important integrators of ERK signalling, in that they direct ERK signalling to or away from the nucleus by acting as dimerisation sites for ERK activation [62] reviewed in [63]. Such a mechanism could explain the role of dystroglycan in integrin-mediated ERK signalling [59], whilst at the same time allowing a dystroglycan-dependent activation of ERK signalling [60] depending on whether the downstream target was nuclear or cytoplasmic [63].

One intriguing possibility that arises from the role of the juxtamembrane region in what appear to be disparate functions: ERM-mediated actin cytoskeleton remodelling and ERK signalling, is the additional observation that the same region is also involved in nuclear targeting of dystroglycan [64]. Whilst the function of dystroglycan in the nucleus and the precise mechanism whereby it is targeted to the nucleus is unresolved it is interesting to consider this in the context of ezrin and ERK, proteins which are also targeted to the nucleus. ERM proteins and the related protein merlin are trafficked to the nucleus in a cell density/and or cell cycle-dependent manner [65,66]. Merlin can regulate the activity of ERK, and ERK and merlin nuclear translocation are stimulated by cell adhesion [65]. Given the interdependence in regulation of ERK by dystroglycan and or merlin (and possibly other ERM proteins) and that the NLS in dystroglycan is also the site of ERM or ERK interaction (Figure 2B) it is possible that ERK or ERM proteins might target dystroglycan to the nucleus but not vice versa. But what is the function of dystroglycan in the nucleus? The observations from the Cisneros lab would suggest there are separate nuclear dystroglycan complexes, containing dystroglycan and the short dystrophin isoform Dp71 and other dystrophin-associated proteins (DAPs), and that these may contribute to the nuclear cytoskeleton or nuclear membrane/cytoskeleton interface [67-69] in a manner analogous to the role of dystroglycan in the sarcolemma. Whether dystroglycan and any complexes it may form in the nucleus have a general role in nuclear architecture as might be suggested by the presence of DAPs, or the presence of dystroglycan in the nucleus is more dynamic and has some sort of regulatory function has yet to be established. More recent evidence from the Cisneros group suggests that there may be dynamic control of dystroglycan nucleocytoplasmic shuttling mediated by importins (Bulmaro Cisneros personal communication), but function still remains to be elucidated.

A further role for the juxta-membrane region of β-dystroglycan is in binding to the neuromuscular junction (NMJ) protein rapsyn [70]. Dystroglycan and rapsyn are essential for the agrin-mediated clustering of acetylcholine receptors at the NMJ [71,72], where they form a specific complex with utrophin even in mature muscle fibres (see [73] and reviews in [74,75]). The RING-H2 domain of rapsyn appears to be responsible for the interaction with dystroglycan [76], and clearly given the separation from the C-terminal WW binding motif to which utrophin could bind, these two interactions could be simultaneous. But how the rapsyn-dystroglycan interaction in this region is accommodated in the face of potential competing interactions between ezrin and ERK remains to be tested. Furthermore, whilst binding to rapsyn is not in question, a role for ERM proteins in mediating acetylcholine receptor clustering through the same site has not been ruled out. Another dystroglycan interaction that has been characterised at the molecular level also occurs at synapses, though in this instance in the hippocampus. S-SCAM, also known as MAGI-2, itself a scaffold protein in inhibitory synapses in the hippocampus [77], interacts with dystroglycan at the N-terminal type 1 WW domain interaction motif [78] (Figure 2). To date S-SCAM is the only protein to be identified to bind to this region of dystroglycan. The precise role of dystroglycan in binding to S-SCAM, and how this impacts on S-SCAM functions as a scaffold to activate RhoA protein in response to NMDA receptor signaling in dendrites [79], is not clear.

## Conclusions

**Figure 5:**
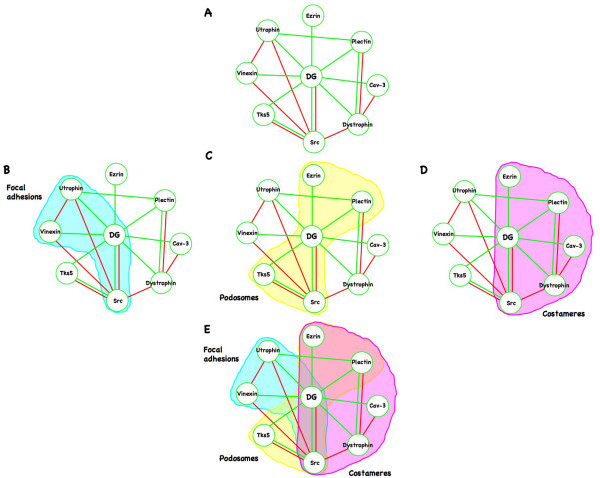
**Interaction map for some of the dystroglycan cytoplasmic domain interacting proteins discussed in this review**. A. Individual named proteins are represented as circles (caveolin-3; Cav-3, dystroglycan; DG) with binding interactions between each them represented by green lines. Competing and regulatory interactions or mutually exclusive interactions are represented by red lines, e.g. phosphorylation of DG by Src prevents utrophin or dystrophin binding so a red line is drawn between Src and utrophin or dystrophin, Caveolin-3 and dystrophin both bind to and compete for the same site on DG, so a red line is drawn between them. B-D represents clusters of interactions that are more specific to the different types of adhesion structure mentioned in the text. Focal adhesion interactions are outlined in blue (B), podosome interactions are outlined in yellow (C) and costameric interactions in magenta (D). E shows an overlay of all of these interaction groups and highlights the central role of the DG-Src axis in acting as a molecular switch to control the various functions and interactions of dystroglycan.

From our own studies and those of others it is clear that dystroglycan can form a range of multiprotein complexes in the cell; as a component of adhesions in the DGC of skeletal muscle, in focal adhesions and in podosomes, as a scaffold and regulator for the ERK MAP kinase cascade, as a dystroglycan-Dbl-ezrin complex involved in the regulation of the actin cytoskeleton, and as yet to be defined role in the nucleus (Figure 4). The role of dystroglycan and how it is regulated both spatially and temporally to maintain these apparent separate functions remains to be fully elucidated. One clear mechanism of modulation is through tyrosine phosphorylation acting as a molecular switch to regulate different binding partners for dystroglycan and hence control its function. In this regard the dystroglycan-Src axis appears to be central to the control of dystroglycan function at least as far as cell adhesion is concerned (Figure 5 and additional file 1). There is still much to learn about this enigmatic protein. One emerging area is the role of dystroglycan in cancer. Dystroglycan function appears to be disrupted in numerous epithelial derived cancers [9,80,81] though the precise functional role of dystroglycan in this context is not clear, but probably involves aspects of both cell adhesion and cell migration, and possibly its putative nuclear function. Furthermore, studies in Drosophila have revealed a role for dystroglycan in both apico-basal and anterior-posterior cell polarity [43,82-84]. It is therefore very likely that the multifunctional adaptor role of the dystroglycan cytoplasmic region will lead to the identification of other interactions and distinct dystroglycan complexes in others systems.

## Competing interests

The authors declare that they have no competing interests.

## Authors' contributions

SJW wrote the initial draft of the manuscript and CJM revised it critically for important intellectual content; and both have given final approval of the version to be published.

## Supplementary Material

Additional file 1 **Animated Powerpoint version of Figure **5. Interaction map for some of the dystroglycan cytoplasmic domain interacting proteins discussed in this review. A. Individual named proteins are represented as circles (caveolin-3; Cav-3, dystroglycan; DG) with binding interactions between each them represented by green lines. Competing and regulatory interactions or mutually exclusive interactions are represented by red lines, e.g. phosphorylation of DG by Src prevents utrophin or dystrophin binding so a red line is drawn between Src and utrophin or dystrophin, Caveolin-3 and dystrophin both bind to and compete for the same site on DG, so a red line is drawn between them. Focal adhesion interactions are outlined in blue, podosome interactions are outlined in yellow and costameric interactions in magenta. The overlay of all of these interaction groups highlights the central role of the DG-Src axis in acting as a molecular switch to control the various functions and interactions of dystroglycan. Click here for file
