# Supplementary material for: Dystroglycan versatility in cell adhesion: a tale of multiple motifs
Source: Cell Commun Signal. 2010 Feb 17;8:3. doi: 10.1186/1478-811X-8-3 (PMC2834674; doi:10.1186/1478-811X-8-3)
Supplement: Additional file 1 — Animated Powerpoint version of Figure 5. Interaction map for some of the dystroglycan cytoplasmic domain interacting proteins discussed in this review. A. Individual named proteins are represented as circles (caveolin-3; Cav-3, dystroglycan; DG) with binding interactions between each them represented by green lines. Competing and regulatory interactions or mutually exclusive interactions are represented by red lines, e.g. phosphorylation of DG by Src prevents utrophin or dystrophin binding so a red line is drawn between Src and utrophin or dystrophin, Caveolin-3 and dystrophin both bind to and compete for the same site on DG, so a red line is drawn between them. Focal adhesion interactions are outlined in blue, podosome interactions are outlined in yellow and costameric interactions in magenta. The overlay of all of these interaction groups highlights the central role of the DG-Src axis in acting as a molecular switch to control the various functions and interactions of dystroglycan. [file 1478-811X-8-3-S1.PPT]

## Slide 1
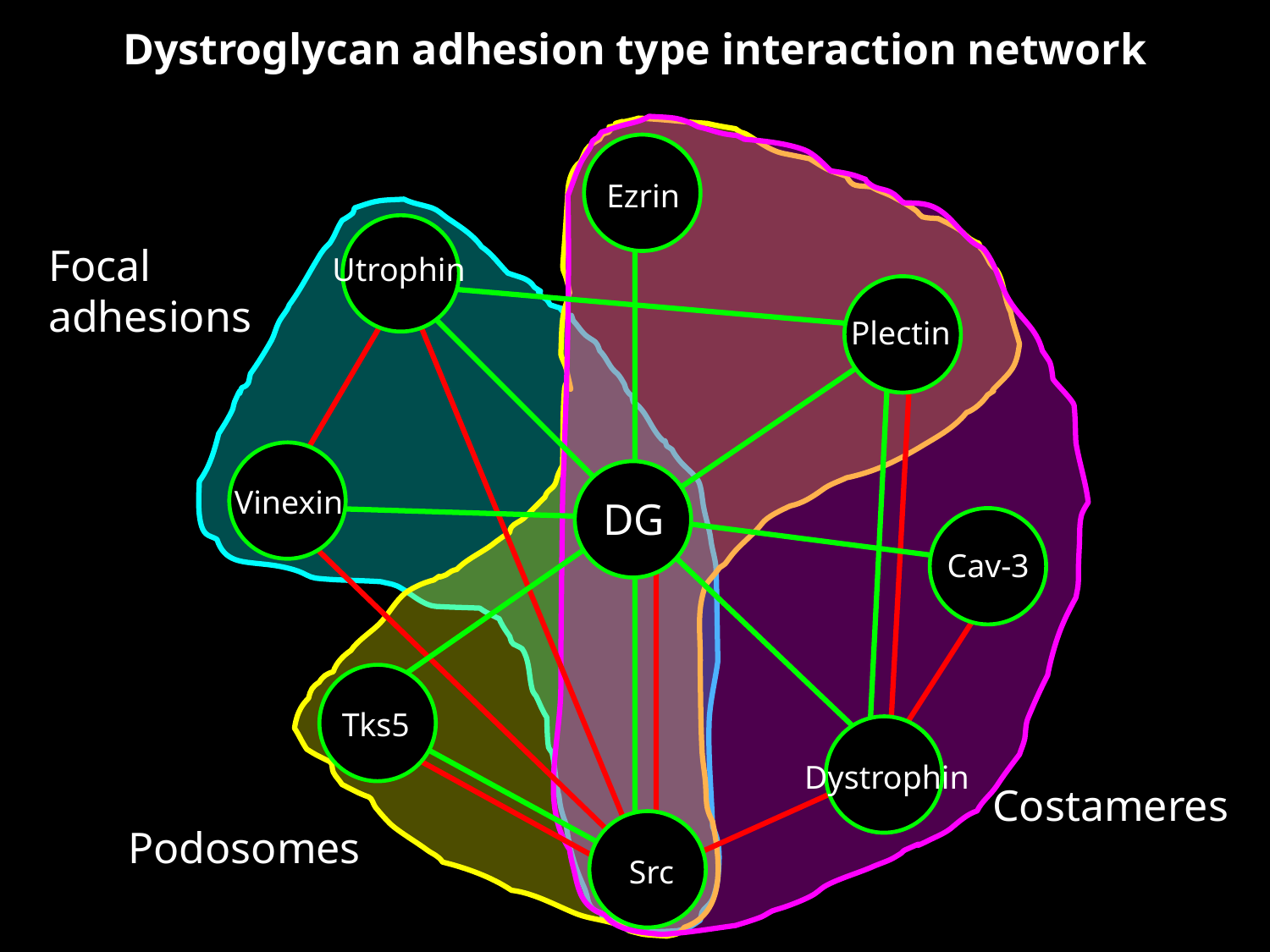

# Dystroglycan adhesion type interaction network
Ezrin
Utrophin
Plectin
Vinexin
DG
Cav-3
Tks5
 Dystrophin
Src
Costameres
Podosomes
Focal
adhesions
